# The acquisition of humoral immune responses targeting *Plasmodium falciparum* sexual stages in controlled human malaria infections

**DOI:** 10.3389/fimmu.2022.930956

**Published:** 2022-07-18

**Authors:** Roos M. de Jong, Manon Alkema, Tate Oulton, Elin Dumont, Karina Teelen, Rie Nakajima, Rafael Ramiro de Assis, Kathleen W. Dantzler Press, Priscilla Ngotho, Kevin K.A. Tetteh, Phil Felgner, Matthias Marti, Katharine A. Collins, Chris Drakeley, Teun Bousema, Will J.R. Stone

**Affiliations:** ^1^ Department of Medical Microbiology and Radboud Centre of Infectious Diseases, Radboud University Medical Centre, Nijmegen, Netherlands; ^2^ Department of Immunology and Infection, London School of Hygiene and Tropical Medicine, London, United Kingdom; ^3^ Department of Physiology and Biophysics, School of Medicine, University of California, Irvine, CA, United States; ^4^ Department of Medicine, Stanford University, Stanford, CA, United States; ^5^ Wellcome Centre for Integrative Parasitology, Institute of Infection, Immunity and Inflammation, University of Glasgow, Glasgow, United Kingdom

**Keywords:** malaria, *Plasmodium falciparum*, sexual stage, gametocyte antigens, antibody responses, controlled human malaria infection (CHMI)

## Abstract

Individuals infected with *P. falciparum* develop antibody responses to intra-erythrocytic gametocyte proteins and exported gametocyte proteins present on the surface of infected erythrocytes. However, there is currently limited knowledge on the immunogenicity of gametocyte antigens and the specificity of gametocyte-induced antibody responses. In this study, we assessed antibody responses in participants of two controlled human malaria infection (CHMI) studies by ELISA, multiplexed bead-based antibody assays and protein microarray. By comparing antibody responses in participants with and without gametocyte exposure, we aimed to disentangle the antibody response induced by asexual and sexual stage parasites. We showed that after a single malaria infection, a significant anti-sexual stage humoral response is induced in malaria-naïve individuals, even after exposure to relatively low gametocyte densities (up to ~1,600 gametocytes/mL). In contrast to antibody responses to well-characterised asexual blood stage antigens that were detectable by day 21 after infection, responses to sexual stage antigens (including transmission blocking vaccine candidates Pfs48/45 and Pfs230) were only apparent at 51 days after infection. We found antigens previously associated with early gametocyte or anti-gamete immunity were highly represented among responses linked with gametocyte exposure. Our data provide detailed insights on the induction and kinetics of antibody responses to gametocytes and identify novel antigens that elicit antibody responses exclusively in individuals with gametocyte exposure. Our findings provide target identification for serological assays for surveillance of the malaria infectious reservoir, and support vaccine development by describing the antibody response to leading vaccine antigens after primary infection.

## Introduction

Gametocytes are the only life stage of *Plasmodium falciparum* that can initiate successful infection in anopheline mosquitoes. The human infectious reservoir in malaria endemic areas is therefore defined by the presence of mature male and female gametocytes in the blood. Interventions to reduce this reservoir or prevent transmission by direct interference with sexual stage development inside mosquitoes could facilitate efforts to achieve malaria elimination (1, 2).

From the point of erythrocyte invasion by a sexually committed merozoite it takes 10 to 12 days for *P. falciparum* gametocytes to fully mature; during this time, they pass through five distinct developmental forms (stages I-V). Immature gametocytes sequester primarily in the bone marrow and spleen outside the peripheral circulation (3–5). They are released back into the circulation to fully mature, after which they can be transmitted to mosquitoes during a blood meal. In the mosquito midgut, gametocytes egress from the host cell, and differentiate into male and female gametes that rapidly undergo fertilisation. In humans, intact immature gametocytes produce proteins that are exported to the erythrocyte surface and elicit an immune response (6). Early reports suggest that naturally acquired antibodies can directly affect gametocyte morphology and maturation, and as a result these antibodies may be able to affect gametocyte numbers and time in circulation (7, 8). Recent evidence indicates that antibodies specific to putative immature gametocyte erythrocyte surface antigens may promote phagocytosis (6), but to what extent gametocytes are specifically targeted and killed in circulation remains unclear. In contrast, there is abundant evidence that immune responses to intra-erythrocytic gametocyte proteins can inhibit gamete fertilisation in the mosquito midgut, when gametes are exposed to the blood meal content after egress from the Red blood cell (9). These target antigens form the basis of advanced transmission blocking vaccines (10).

At present, little is known about antibodies specific for gametocyte proteins, besides the well-characterised gamete fertility proteins (Pfs48/45 and Pfs230). Studies indicate that these proteins may not be the sole contributors to natural transmission blocking immunity (11), so there is an imperative to investigate immune responses to a wider sexual stage protein catalogue. The use of gametocyte specific antibodies as biomarkers of gametocyte carriage and infectiousness may also help identify the infectious reservoir in population-wide surveillance. Prior studies that identified gametocyte-enriched or specific proteins used proteomic data, without reference to immunogenicity (11, 12). Studies assessing anti-gametocyte antibody responses have focused on naturally exposed populations and are thus complicated by lack of effective controls and inherent variance in prior parasite exposure. Controlled human malaria infection (CHMI) models (13) in which malaria naïve volunteers are deliberately infected with *P. falciparum* parasites, provide a powerful tool to study immune responses during a well-characterized primary infection. Classical CHMI does not allow for evaluation of interventions affecting transmission, as gametocytes arise approximately 10 days (14) after asexual parasitaemia peaks, by which time participants have received full curative treatment that does not allow for gametocyte development. Recently, the CHMI model has been adapted to allow safe induction of gametocytes in study participants (12, 15, 16). In these models, volunteers were infected with *P. falciparum* 3D7 parasites and sub-curative treatment of asexual parasites allowed the development of viable mature male and female gametocytes. Infection by injection of infected red blood cells appeared to induce higher gametocyte densities and a higher likelihood of infecting mosquitoes, compared to infection through mosquito bites (16) and resulted in lower inflammation (17).

Here, we assessed antibody responses to sexual stage antigens among participants of a CHMI transmission study (16) after a single induced infection. We examined the immunogenicity of gametocyte proteins, the acquisition of gametocyte-specific antibodies and their association with preceding gametocyte exposure. Bead-based antibody assays allowed us to assess antibody responses to sub-units of the transmission blocking vaccine antigens Pfs48/45 and Pfs230, with comparison to well characterised antibody biomarkers of blood stage infection. Using a protein microarray, we set out to identify novel antigens that are targeted by antibodies uniquely induced after gametocyte exposure, which is of value in the context of serological assay development for gametocyte surveillance.

## Materials and methods

### Clinical trial samples

Samples were collected in a CHMI transmission trial conducted between May and November 2018 (16). Individuals were infected either by the bites of 5 P*. falciparum* 3D7 infected mosquitoes (SPZ Gct, n =12), or by intravenous injection with ~2,800 *P. falciparum* 3D7 infected human erythrocytes (BS Gct, n=12). Parasitaemia was monitored by 18s quantitative polymerase chain reaction (qPCR); after parasitaemia reached a prespecified treatment threshold, participants received a gametocyte permissive sub-curative dose of piperaquine (480 mg) (16). Serum and citrate plasma samples were collected on prespecified time points prior to and after challenge infection. Plasma samples were selected for analyses from blood samples taken prior to challenge (C-1), and at days C+7 (BS Gct), C+9 (SPZ Gct), C+21 and C+36 in BD Cell Preparation Tubes with sodium citrate. One serum sample was selected from day C+51, which was collected in BD SST™ II Advance tubes.

As a control for gametocyte exposure in our antibody assays, additional plasma samples were analysed from control participants in a CHMI study where no gametocyte exposure was anticipated. Although gametocyte exposure was deemed highly unlikely due to early curative treatment, the absence of gametocytaemia was not formally demonstrated. This study was conducted between April 2011 and March 2012 (18); volunteers were infected by mosquito bite (SPZ Control: 5 P*. falciparum* 3D7 infected mosquitoes, participants n=5) or an intravenous blood stage injection (BS Control: 1,962 *P. falciparum* 3D7 infected erythrocytes, participants n=5) and treated with a standard curative regimen of atovaquone/proguanil upon thick smear positivity. Here, we analysed antibody responses in 5 volunteers infected by mosquito bite (SPZ Control, acting as a control for SPZ Gct) and 5 infected with blood stages (BS Control, acting as a control for BS Gct). Plasma samples were collected prior to challenge (C-1), and at days C+7 (BS Control only), C+9 (SPZ Control only), C+21 and C+36 in BD Cell Preparation Tubes with sodium citrate. Plasma samples were collected in BD Cell Preparation Tubes with sodium citrate.

Asexual parasite densities were determined by 18S qPCR on prespecified timepoints as described previously (16, 18, 19). Gametocyte densities were determined using quantitative reverse-transcriptase polymerase chain reaction (qRTPCR) for *ccp4* (female) and *pfmget* (male) messenger RNA, with a limit of detection of 0.1 male or female gametocyte/µL (20).

Both trials were performed at the Radboud university medical centre (Nijmegen, the Netherlands) following approval by the central committee on research involving human subjects (CCMO) under NL34273.091.10 and NL63552.000.17. All study participants provided written informed consent and both trials were registered at clinicaltrials.gov under NCT01236612 and NCT03454048. Antibody responses were specified as an exploratory outcome measure in the CHMI transmission study; the current analyses are thereby ancillary to the main study evaluation that focused on safety, gametocyte density and infectivity.

### Gametocyte and asexual ELISA

Gametocyte and asexual extracts were prepared as described previously (21). Nunc MaxiSorp™ 96-wells plates (ThermoFisher) were coated overnight at 4⁰C with 100 µl, equivalent to 75,000 gametocytes or 40,000 asexual parasites, per well. Plates were blocked with 5% skimmed milk in PBS and subsequently incubated with an 1:50 dilution of citrate plasma. Detection was done with 1:40,000 dilution Goat anti-Human IgG HRP (Invitrogen, Cat. No. **31412).** ELISAs were developed by adding 100 µL tetramethylbenzidine and stopped with 50 µL 0.2M H_2_SO_4_. Absorbances were read at 450nm on an iMark™ microplate absorbance reader (Bio-Rad).

ELISA analyses were performed using Auditable Data Analysis and Management System for ELISA (ADAMSEL FPL v1.1). We included serial diluted control serum from a Dutch missionary that experienced many malaria episodes as a standard curve. The standard curve was plotted on a logarithmic scale and fitted to a power trend line (R2> 0.99), optical density (OD) measurements for each test sample (average of duplicates that were no more than 25% different) were converted to arbitrary units (AU) relative to the control serum, where undiluted control serum was defined to contain 100 AU of IgG.

### Bead-based antibody quantification

IgG antibodies against 21 antigens, one targeting pre-erythrocytic stages (Circumsporozoite protein [CSP] (22)), 15 targeting the asexual blood stage (Erythrocyte binding antigen [EBA140, EBA175 and EBA181] (23); Early transcribed membrane protein 5 [Etramp-5] (24); Glutamate rich protein 2 [GLURP-R2] (25); Heat shock protein 40 [HSP40] (24); Merozoite surface protein 1-19 [MSP1-19] (26), Merozoite surface protein 2 [MSP2-ch150/9 (3D7 family allele) (27), and MSP2-DD2 (FC27 family allele) (28); Schizont egress antigen 1 [SEA-1] (29); Skeleton binding protein 1 [SBP-1] (30); Apical membrane antigen 1 [AMA1] (31); and Reticulocyte binding protein homologue [RH2.2 (32), RH4.2 (33), RH5.1 (34)] and five (belonging to 2 proteins) targeting sexual stages [four fragments of Pfs48/45; Pfs230-CMB (35)] were quantified for all samples for each participant using a Luminex MAGPIX^©^ suspension bead array, as described previously (36). The proper conformation of Pfs48/45 recombinant proteins was validated using conformational dependent rat monoclonal antibodies (37). A complete list of antigens is provided in **Table S1**. Briefly, plasma/serum samples were assayed at a dilution of 1:200. Secondary antibody was an R-phycoerythrin conjugated goat anti-human IgG (Jackson Immuno Research, PA, USA; 109-116-098) diluted to 1:200. Data are presented as background adjusted median fluorescence intensities (MFI), or the same measure as a log2 ratio of each individual’s adjusted MFI at baseline.

### Protein microarray

*De novo* protein microarrays were designed and printed at the University of California, Irvine, to assess antibody responses to a panel of gametocyte enriched *P. falciparum* proteins. The backbone for protein selection was an analysis of specificity for the gametocyte stage, as scored by determining frequency of detection across 11 proteomic analyses. This analysis is described in detail elsewhere (12). In summary: Proteins were binned from low to high abundance and weighted according to the retrieval rates of proteins in two curated lists of ‘gold standard’ gametocyte and asexual genes, consisting of genes that are known to be specific for either asexual stages (n = 45) or gametocytes (n = 41). High expression of gametocyte gold standard proteins with concurrent absence of non-gametocyte gold standard proteins resulted in a high gametocyte score, calculated from the fraction of retrieved gametocyte genes over retrieved non-gametocyte genes. All scores were log-transformed and summed over all data sets.

Full criteria for inclusion on the array are presented in **Table S2**. In addition to gametocyte specificity, proteins were prioritized according to their likelihood of gamete surface expression (based on gene ontological terms, domain prediction, or empirical evidence), or association with gametocyte exposure (38), transmission blocking immunity (39) or antibody recognition of immature gametocyte infected red blood cell (giRBC) surfaces (6). In total, a selection of 600 unique *P. falciparum* genes were selected for cloning. Sequences encoding the proteins were obtained from a 3D7 strain reference genome, with sequences longer than 1000 amino acids split into multiple fragments (overlaps of at least 17 amino acids). PCR amplification and cloning were successful for 568 unique genes (making up 943 distinct sequences) all of which were expressed in an *in vitro* transcription and translation (IVTT) system (5 Prime, Gaithersburg, MD, USA) according to manufacturer instructions, and as described previously (39, 40). Arrays were printed onto 8-pad nitrocellulose-coated glass AVID slides (Grace Bio-Labs, Inc., Bend, OR, USA) using an Omni Grid Accent robotic microarray printer (Digilabs, Inc., Marlborough, MA, USA). Each array and subarray contained IgG positive controls, for quality control, and negative controls containing the products of the IVTT reaction without PCR vector, for data normalisation. Samples were processed as described previously, with small deviations. Samples were probed at a final dilution on of 1:200, with secondary antibody (Southern Biotech, Goat Anti-Human IgG-TXRD) at a concentration of 0.5 µg/mL (39). Arrays were scanned on a GenePix 4300A High-Resolution Microarray Scanner (Molecular Devices, Sunnyvale, CA, USA). Local background was assessed for each protein target automatically, with foreground MFI determined using irregular threshold pixel density mapping. Correction for background was done for each spot using the ‘backgroundCorrect’ function of the *limma* package (41). Background corrected values were transformed using the base 2 logarithm and normalised to systematic effects by subtracting the median signal intensity of the negative IVTT controls (internally within four subarrays per sample). The final normalised data are a log2 MFI ratio relative to the background reactivity of each sample/sub-array: a value of 0 represents equality with the background, and a value of 1 indicates a signal twice as high.

### Data analysis

Data analysis was performed using R (R foundation for statistical computing, Vienna, Austria; version 4.1.2) (42), STATA (StataCorp. 2021. College Station, TX: StataCorp LLC; Release 17), or Graphpad PRISM (Graphpad software, San Diego, CA, USA; version 8). Total parasite and gametocyte area under the curve (AUC) was computed using GraphPad prism software with the formula AUC = (ΔX)*(Y1 + Y2)/2, where X is the time in days and Y the parasite density at a given timepoint. Correlation between parasite and serological data were analysed by Spearman’s rank order correlation. For the analysis of breadth in the purified antigen antibody assays, antigens were considered ‘recognised’ for a given timepoint and participant if an antibody response (log transformed background adjusted MFI) exceeded the mean plus 2*SD of all individuals (n=34) against the same antigen pre-challenge. For the analysis of breadth in the protein microarray antibody assays, antigen recognition was defined as any log2 MFI ratio value greater than 1 (double the signal with respect to the internal array control). Serological data were log transformed and compared between timepoints within cohorts by paired t-tests, and between cohorts by students t-tests. For recombinant protein assays, significance thresholds were adjusted by Bonferroni correction for comparisons of multiple antigens. Comparison between array responses between timepoints and cohorts were conducted with bayes moderated t-tests with adjustment for false discovery (43).

## Results

In the CHMI transmission trial, 12 participants were infected by mosquito bite (SPZ Gct) and 12 participants were infected by intravenous injection of asexual parasites (BS Gct) (16). All participants developed parasitaemia with median onset of qPCR detectable parasitaemia on day 7 (Interquartile range (IQR) day 7 – 9) in SPZ Gct and on day 5 (IQR day 5 – 5) in BS Gct (**Figure S1**). To clear asexual parasitaemia but permit gametocyte maturation, participants received treatment with low dose piperaquine (480mg) on day 12.25 (Median, Interquartile range (IQR) day 10.5 – 12.5) in SPZ Gct, and all BS Gct participants were treated on day 8. This resulted in qRT-PCR detectable gametocytes post treatment in 11/12 participants in SPZ Gct and 12/12 participants in BS Gct (16).

In the control CHMI cohorts, 5 participants were infected by mosquito bite (SPZ Control) and 5 participants were infected by intravenous injection of asexual parasites (BS Control) with the same *P. falciparum* 3D7 parasite clone as the CHMI transmission trial. In contrast to the CHMI Gct trial, participants from control cohorts received a full curative treatment with atovaquone/proguanil that does not permit gametocyte maturation, initiated on day 12.3 (Median, IQR day 9.8 – 12.3) in SPZ Control and on day 8 (IQR day 8 - 8) in BS Control. As such, these participants were included in our analyses as negative controls without gametocyte exposure (**Table 1**), although there was no formal demonstration of gametocyte negativity.

**Table 1:** Parasitaemia and gametocytaemia for different cohort.

		Mosquito bite (sporozoite) infection		Asexual stage infection	
		SPZ Control	SPZ Gct	BS Control	BS Gct
Total parasites	Median peak density in parasites/μL (IQR)	7,413 (2,336 – 28,371)	32,807 (7,137 – 50,831)	44,668 (27,184 – 74,505)	27,700 (9,818 – 81,091)
	Median AUC in parasites/μL/day (IQR)	8,682 (2,782 – 29,890)	37,654 (15,430 – 71,484)	62,470 (39,606 – 103,950)	38,735 (11,366 – 75,145)
Gametocytes	Median peak density in gametocytes/μL (IQR)	ND	14 (10 – 64)	ND	1,304 (308 – 1,607)
	Median AUC in gametocytes/μL/day (IQR)	ND	1,574(596 – 3,018)	ND	11,043 (2,715 – 14,866)

AUC, Area under the curve; IQR, Interquartile range; ND, Not determined.

Neither peak total parasite density nor AUC were significantly different between SPZ Gct and SPZ Control (peak density, p=0.091; AUC, p=0.058), and between BS Gct and BS Control (peak density, p=0.673; AUC, p=0.206) or between SPZ Gct and BS Gct (peak density, p=0.478; AUC, p=0.977). However, significantly higher peak gametocyte densities were observed in BS Gct compared to SPZ Gct (p<0.001, Mann-Whitney U; **Table 1**) (16). Total parasite AUC and gametocyte specific AUC were positively associated for both SPZ Gct and BS Gct (**Figure S2A**); a tighter correlation was observed for BS Gct, reflecting the controlled timing of blood stage infection and subsequent schizogony in this cohort.

### Antibody response to crude gametocyte extract does not reflect preceding gametocyte exposure

**Figure 1:**
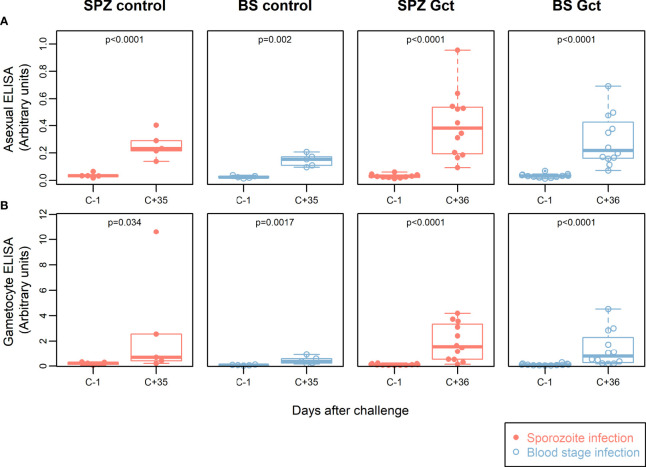
Participant parasite exposure and antibody response to crude parasite extracts. IgG antibody responses to crude asexual and gametocyte extracts. In all plots, red solid circles denote mosquito bite (sporozoite) infection cohorts (SPZ Control, SPZ Gct) and blue hollow circles denote asexual parasite infection (BS Control, BS Gct). **(A)** Anti-asexual antibody response pre- and post- (day 36) infection. **(B)** Anti-gametocyte antibody response pre- and post- (day 36) infection. p = p-values from paired t-tests on log-transformed data.

Extracts from mixed asexual stage and mature gametocytes from laboratory cultured *P. falciparum* NF54 were prepared to assess antibody responses in the CHMI participants to native parasite proteins (**Figure 1**). At C+35/36 after infection, a statistically significant increase in anti-asexual stage antibodies was found compared to baseline in both SPZ Gct and BS Gct (p<0.0001) and corresponding SPZ Control (p<0.0001) and BS Control (p=0.002). Anti-asexual stage IgG antibody levels post infection were not statistically different between infection routes SPZ Gct and BS Gct (p=0.22), and borderline significant between SPZ Control and BS Control (p=0.050).

IgG levels against gametocyte extract were also significantly higher post CHMI compared to baseline in the SPZ Gct and BS Gct groups (p<0.0001). There was no statistically significant difference in antibody responses to gametocyte extract between SPZ Gct and BS Gct cohorts (p=0.31). Although participants in control cohorts were not exposed to sexual stage parasites during their CHMI, some subjects infected by mosquito bites (SPZ Control) showed an increase in anti-sexual stage IgGs in response to infection. On average, antibody responses to gametocyte extract were higher after infection for both control cohorts (SPZ Control, p=0.034, BS Control, p=0.0017), though for SPZ Control this increase was disproportionally driven by a single volunteer (after exclusion, p=0.075).

There were no statistically significant correlations between total parasite AUC or gametocyte AUC and either asexual or gametocyte antibody responses by ELISA (**Figures S2B,C**).

### Sexual stage-specific antibodies are induced after limited gametocyte exposure

We observed that antibody responses to crude gametocyte extracts cannot be used to discriminate between responses to asexual and sexual parasite stimuli. This indicates, perhaps unsurprisingly, an abundance of proteins of unknown stage-specificity in the gametocyte extract that formed the basis of this assay. We therefore analysed antibody responses to 21 well-characterized asexual stage, sporozoite, and sexual stage recombinant proteins, including TBV candidates Pfs48/45 and Pfs230, in a multiplexed bead-based antibody assay.

At baseline, the median number of antigens recognised per individual in SPZ Gct and BS Gct were 0.5 and 0, respectively (**Figure S3**). By C+36, all individuals were seropositive to at least one antigen, with a statistically significant increase in antibody breadth in SPZ Gct (p = 0.025), but not BS Gct (p = 0.12). At C+51, 21/21 antigens were recognised by at least one individual in both cohorts. Between C+36 and C+51, there was no significant increase in breadth of response in SPZ Gct (p = 0.23), but a significant increase in BS Gct (p = 0.015). Breadth of antigen recognition in the control cohorts was similar to the Gct groups at relative time points; the increase in breadth scores from baseline to C+35 was statistically significant in both SPZ Control (p = 0.041) and BS Control (p = 0.0086) and the change in median score was equal between control groups (SPZ Control, change in median = +2; BS Control, change in median = +2). In contrast, the increase in the median number of antigens recognized in the Gct groups over the same time period was nearly twice as great in SPZ Gct (change in median = +3.5) compared to BS Gct (change in median +2).

**Figure 2:**
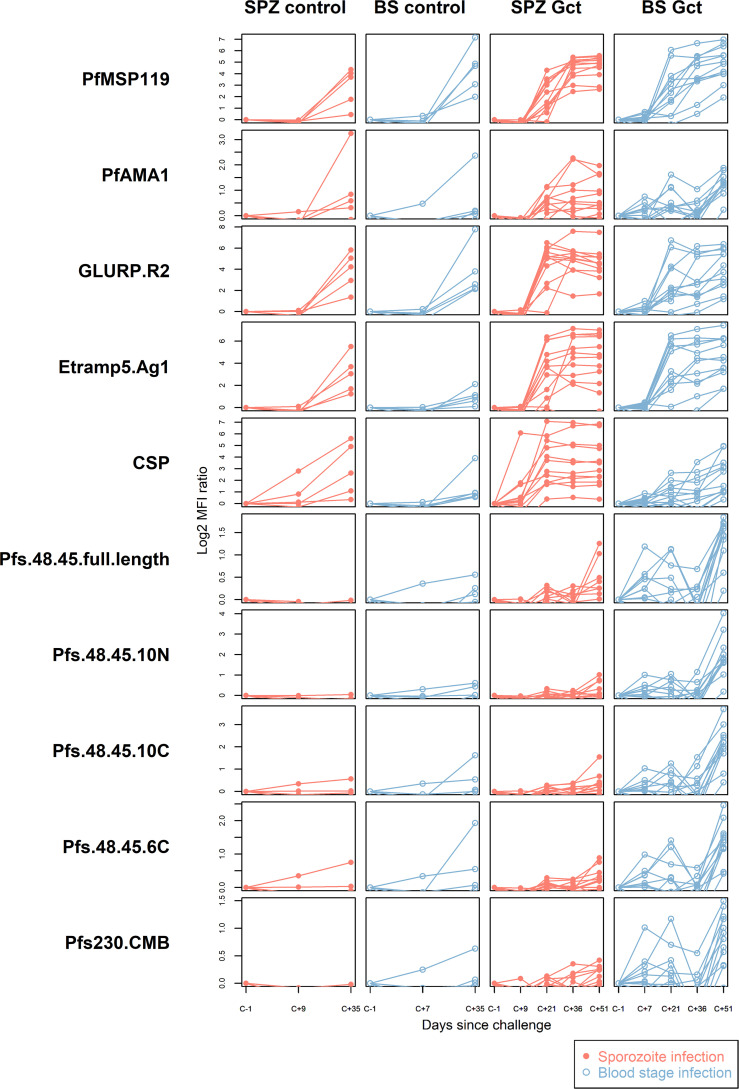
Antibody responses to purified recombinant proteins. IgG antibody responses to a selection of purified sporozoite, asexual and sexual stage antigens. Statistical analysis of response over time compared to baseline for all antigens is in **Supplemental Table S3**. In all plots, red solid circles denote mosquito bite (sporozoite) infection cohorts (SPZ Control, SPZ Gct) and blue hollow circles denote asexual parasite infection (BS Control, BS Gct). Data are presented as log2 median fluorescence intensity (MFI) ratios of response over baseline, each line representing a single individual.

As observed in the cell extract assays, quantitative antibody responses to specific recombinant antigens increased significantly over the period of observation (**Figure 2**). In the Gct cohorts, the earliest independently significant increases in antibody response were observed at C+21 for a small number of non-sexual stage antigens (**Table S3**). However, after adjustment for multiple comparisons the only significant response observed at day 21 was against CSP in the SPZ Gct cohort (p=0.001). At C+35/36 a greater number of antigens showed increased responses, with those to PfMSP1-19 in SPZ Gct and BS Gct, and GLURP-R2 in SPZ Gct remaining significant after adjustment for false discovery. At C+51, several asexual antigens remained significantly elevated including ETRAMP5 and GLURP-R2 in SPZ Gct, EBA175, MSP2-DD2 and PfAMA-1 in BS Gct, and PfMSP1-19 in both cohorts. After adjustment for false discovery, antibody responses to the sexual stage antigens Pfs230, Pfs48/45 full length, and the 10C fragment of Pfs48/45 were all significantly higher at C+51 compared to baseline in BS Gct, but not SPZ Gct. No statistically significant antibody responses to gametocyte antigens were observed in SPZ Gct at any point, or in either cohort before C+51.

In the control cohorts, independently significant responses were observed for HSP40 in SPZ Control, and EBA140 and PfMSP1-19 in BS Control; these increases were not statistically significant after adjustment for false discovery. It should be noted that samples were only available until C+36 for these cohorts.

### Responses to gametocyte antigens occur after responses to asexual antigens

To determine possible shifts in antibody responses during follow-up, supportive of responses specifically induced by gametocytes, we next compared antibody responses and their relative rankings between days C+35/36 and C+51. For this, antibody responses to all antigens were ranked by median magnitude of response in each cohort (**Figure S4**) and compared between C+35/36 and C+51 timepoints.

Within the CHMI Gct cohorts, median magnitude-ranked responses to sexual stage antigens were most increased between C+36 and C+51 in BS Gct, with Pfs48/45-10C, Pfs48/45-10N and Pfs48/45 full length moving up by 5, 4 and 5 positions, respectively (**Table S4**). In SPZ Gct, Pfs48/45-10C and Pfs48/45-10N moved up by 1 and 2 positions, though Pfs48/45 full length fell by 1 position. Little to no change was observed for Pfs48/45-6C or Pfs230-CMB in either cohort between timepoints. Sexual stage antigen rankings at C+35/36 between SPZ Gct and SPZ Control were similar, while median responses tended to rank higher in BS Control than in BS Gct at the equivalent timepoints.

### Antibody response to gametocyte infected erythrocyte surface antigens are among those correlated with cumulative gametocyte exposure

The correlation between each recombinant protein biomarker and asexual or gametocyte exposure was assessed by analysing antigen-specific antibody data from the final timepoint of observation (C+51) in the CHMI Gct cohorts (**Figure S5**, **Table S5**) in relation to prior parasite biomass (i.e. area under the curve of density over time). In SPZ Gct, responses to Pfs230 and Pfs48/45-10N were independently correlated with asexual and gametocyte AUC, but these correlations were not statistically significant after adjustment for multiple comparisons. For BS Gct, several asexual and sexual stage responses were independently correlated with asexual and gametocyte AUC; after adjustment only PfMSP1-19 showed a statistically significant positive correlation with asexual AUC (R^2 =^ 0.64, p=0.0017), and only Pfs48/45-10C showed a statistically significant positive correlation with gametocyte AUC (R^2 =^ 0.64, p=0.0019).

**Figure 3:**
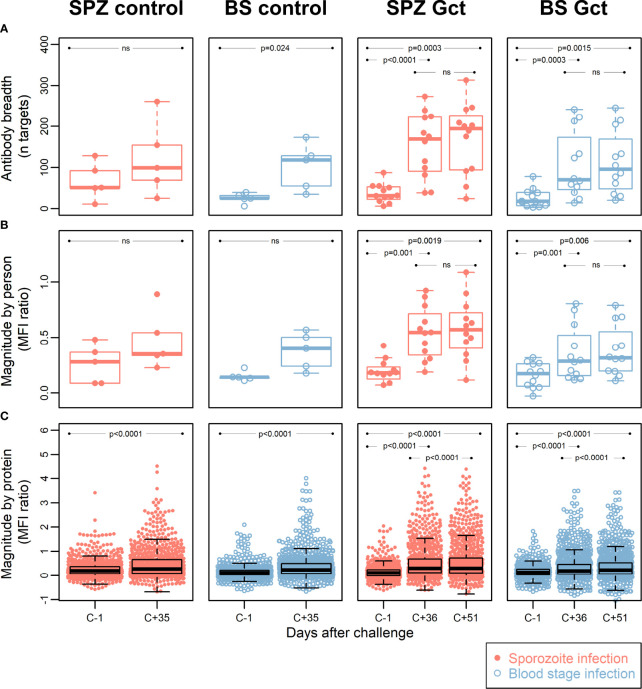
Antibody breadth and magnitude. Data points for each individual within each cohort group are represented with dots in a beeswarm pattern. Overlayed boxplots represent the median (thick line), interquartile range (box limits) and the 25^th^/75^th^ percentiles plus 1.5*IQR (whiskers). **(A)** Antibody breadth is the number of protein targets (out of a total of 943) with responses above background, for each individual. For SPZ Gct, the mean MFI ratio of antibody responses to all proteins for one individual was -0.47 at C-1; this data point was not included in the plot, but the parameters of the relevant box plot were calculated from all data points. **(B)** Antibody magnitude is shown as the mean magnitude of response by each individual in a cohort group/timepoint to all protein targets. Magnitude of response is shown as a log2 MFI ratio, where 0 represent no change relative to background, and 1 represents doubling with respect to background. **(C)** Antibody magnitude is shown as the mean magnitude of response to each protein target by all individuals in a cohort group/timepoint, with units as in **(B)** P-values are from paired two-sided t-tests for difference between C-1 and C+35/36, C-1 and C+51, or C+36 and C+51, as indicated. MFI: median fluorescence intensity; IQR: Interquartile range. P = P-value from paired t-tests. Ns = Not significant at p = 0.05.

To identify novel antibody biomarkers of gametocyte exposure, 943 protein targets (mapping to 568 gene IDs) were printed on microarrays, following selection for their enrichment in gametocyte stages based on transcriptomic and proteomic evidence (**Table S2**), or inclusion as known *Plasmodium* biomarkers. Antibody breadth increased significantly after infection (C+35/36) in all cohorts except for SPZ Control (**Figure 3A**). Mean magnitude of response to all antigens for each participant increased significantly in SPZ Gct and BS Gct, but not in the controls, while a significant increase in mean magnitude of response to each target protein was observed for all cohorts (**Figures 3B**, **C**). Correlation in mean response to each target between the two included post-infection timepoints (C+36 compared to C+51) was near perfect for SPZ Gct (R^2 =^ 0.99, p<0.0001) and BS Gct (R^2 =^ 0.98, p<0.0001).

At the level of individual targets, 216 of the 943 IVTT protein targets on the array displayed a significant increase in antibody response from pre-infection to either C+36 or C+51 in SPZ Gct, and 91 showed a significant increase in BS Gct. Four of the 91 targets with statistically significant responses in BS Gct were uniquely responsive in this cohort: PF3D7_0905300 (dynein heavy chain, putative; 1.65 fold increase compared to baseline), PF3D7_1302000 (EMP1-trafficking protein; 1.31 fold increase), and PF3D7_0721100 (conserved protein, unknown function; 1.32 fold increase), and PF3D7_1306500 (MORN repeat protein, putative, 1.15 fold increase) (**Table S6**). In the control cohorts, the only statistically significant increase in antibody response (C-1 to C+35) after adjustment for multiple comparisons was PF3D7_0206800 (MSP2) in SPZ Control.

**Figure 4:**
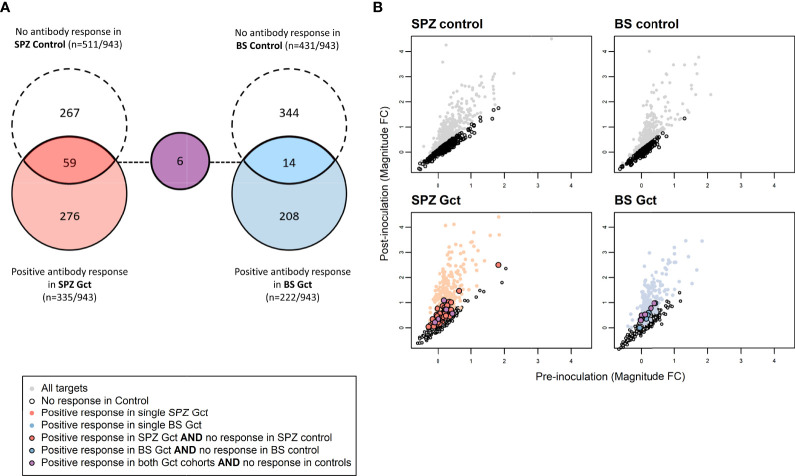
Differences in antibody response to microarray targets associated with gametocyte exposure. **(A)** Array targets demonstrating minimal response (<7.5% increase in the mean magnitude of response between pre- [day 0] and post- [day 35] inoculation timepoints) in the control CHMI cohorts are shown in red for SPZ Control (sporozoite inoculum) and blue for BS Control (asexual inoculum). Array targets demonstrating a positive response (>7.5% increase in the mean magnitude of response between pre- [day 0] and post- [day 36] inoculation timepoints) in the control CHMI cohorts are shown in dashed circles for each cohort, with overlap representing those targets with minimal response in control and positive response in Gct cohorts. **(B)** Axes show pre- and post-inoculation mean magnitude of response (log2 MFI ratio) to each target on the array. Mean magnitude for each target is represented by a single marker. Grey markers are those with increased responses after inoculation in the Control cohorts. Red and blue markers are as in **(A)** Targets with responses only in the CHMI Gct cohorts are outlined in black.

To further distinguish antibody responses elicited by the principally asexual stimulus of the control CHMI studies from responses to the asexual and gametocyte stimulus of the Gct studies, the mean magnitude of response to each target post-infection was compared between control and Gct cohorts according to infection methodology. A threshold for negligible change in response between time points in the control cohorts was set arbitrarily as any percent difference in MFI of 7.5% or less, while a positive response cut-off for the Gct cohorts was set at 25%. 348 targets showed increases in magnitude (>25% absolute fold change) from pre- to post- (C+36 or C+51) infection in the Gct cohorts (**Figure 4**). Of these, 67 were responsive only in the SPZ Gct and/or BS Gct and not in their respective control cohorts; 53 were unique to SPZ Gct (median maximum percent change between cohorts at C+36 or C+51: 37.5% [IQR 30.7-47.4]), 8 were unique to BS Gct (35.0% [30.3-39.0]), and 6 were shared in both (SPZ Gct: 37.1% [32.9-41.6], BS Gct: 38.6 [29.0-44.1]) (**Table S6**). Nineteen targets were uniquely identified in the day 36 analysis, 6 in the day 51 analysis, and 42 were identified as responsive in both. A sub-selection of putative biomarkers of gametocyte exposure was made and is shown in **Table 2**. This includes the four targets described above with significantly higher antibody responses (day C+36 and/or 51) compared to baseline in BS Gct but not SPZ Gct, six with responses in both SPZ Gct and BS Gct but not in their methodological control cohorts, and six with response in either SPZ Gct or BS Gct where a response was observed at C+51 but not at C+36 (two targets met more than one of these criteria). There was no significant correlation between antibody responses to these targets and total parasite AUC in either SPZ or BS Gct (**Figure S6A**), and no correlation between response and gametocyte AUC in SPZ Gct (**Figure S6B**). For BS Gct, 4 targets showed independently significant or borderline significant associations with gametocyte AUC: PF3D7_0721100 (R^2 =^ 0.33, p=0.051; conserved *Plasmodium* protein, unknown function), PF3D7_1302000 (R^2 =^ 0.47, p=0.014; EMP1-trafficking protein [PTP6]), PF3D7_0726400 (R^2 =^ 0.37, p=0.037; conserved *Plasmodium* membrane protein, unknown function), and PF3D7_1016300 (R^2 =^ 0.34, p=0.046; glycophorin binding protein [GBP]). None of these remained significant after adjustment for false discovery.

**Table 2:** Antibody targets putatively linked with higher gametocyte exposure as identified by protein microarray.

Gene ID	Name	Description	Criteria for selection	Note
PF3D7_0905300		dynein heavy chain, putative	*	
PF3D7_1306500		MORN repeat protein, putative	*	Naturally acquired transmission blocking immunity (mosquito stage) (39)
PF3D7_1351000		phosphatidylinositol transfer protein, putative	BS/51***	
PF3D7_1125200		ubiquitin-like domain-containing protein, putative	BS/51***	
PF3D7_0721100		conserved Plasmodium protein, unknown function	BS/51*^/^***	Early giRBC membrane antigen, putative (6)
PF3D7_1409400		conserved Plasmodium membrane protein, unknown function	SPZ/51***	
PF3D7_1135600	CAPD3	condensin-2 complex subunit D3, putative	SPZ/51***	
PF3D7_1016300	GBP130	glycophorin binding protein (GBP)	SPZ/51***	Early giRBC membrane antigen, putative (6)
PF3D7_1328000		conserved Plasmodium protein, unknown function	SPZ and BS/36 and 51**	
PF3D7_0307900		conserved Plasmodium protein, unknown function	SPZ and BS/36 and 51**	
PF3D7_0726400		conserved Plasmodium membrane protein, unknown function	SPZ and BS/36 and 51**	Early giRBC membrane antigen, putative (6) AND Naturally acquired transmission blocking immunity (mosquito stage) (39)
PF3D7_0201900	EMP3	erythrocyte membrane protein 3 (EMP3)	SPZ and BS/36 and 51**	Early giRBC membrane antigen, putative (6)
PF3D7_1320800		dihydrolipoyllysine-residue succinyltransferase component of 2-oxoglutarate dehydrogenase complex	SPZ and BS/36 and 51**	
PF3D7_1302000	PTP6	EMP1-trafficking protein (PTP6)	SPZ and BS/36 and 51*^/^**	Early giRBC membrane antigen, putative (39)

Summary table listing targets with significantly higher antibody responses (day C+36 and/or 51) compared to baseline in BS Gct but not SPZ Gct (*n=4), in both SPZ Gct and BS Gct compared to their methodological control cohorts (**n=6), and in either SPZ Gct or BS Gct where a response was observed at C+51 but not at C+36 (***n=6 [overlapping]). **BS =** Antibody response in blood stage infection cohort, SPZ = Antibody response in sporozoite infection cohort, 36 = Antibody response at C+36 compared to methodology matched control, 51 = Antibody response at C+51 compared to methodology matched control. Full details of these targets and all targets described in the text are given in **Supplemental Table 6**.

The 69 array targets meeting any of our criteria for further investigation (**Table S6**) mapped to 64 unique gene IDs; three IDs were represented by two peptides (PF3D7_1250100; osmiophilic body protein (G377), PF3D7_0212400; conserved *Plasmodium* membrane protein, unknown function, PF3D7_1328000; conserved *Plasmodium* protein, unknown function) and one was represented by three peptides (PF3D7_1038400; gametocyte specific protein Pf11-1). Association with cell membranes was highly represented (n/N=27/65) among predicted gene ontological terms (**Table S6**). Antibody responses to several well-characterised blood stage antigens were associated with gametocyte exposure in our analyses, including PF3D7_1228600 (merozoite surface protein 9, MSP9), PF3D7_0711700 (erythrocyte membrane protein 1, PfEMP1 [VAR]), and PF3D7_0102200 (ring-infected erythrocyte surface antigen, RESA). Well-characterised sexual stage antigens included PF3D7_1302100 (gamete antigen 27/25 [G27/25]), PF3D7_1102500 (parasite/early gametocyte exported protein PHISTB/GEXP02]), and PF3D7_1038400 (gametocyte-specific protein [Pf11-1]). Overall, 4/64 targets were specific to asexual stages, 42 were specific or enriched in gametocytes, 17 showed more evenly shared stage expression, and 1 was unclassifiable. Antibody responses to eleven of the 64 gene products identified here were noted for their association with naturally acquired transmission blocking immunity (TBI) in a previous analysis of individuals from Burkina Faso, Cameroon and Gambia (39). Nine of the 64 genes (three also linked with TBI) were identified as putative early gametocyte erythrocyte surface antigens in a previous analysis of rodent infections and sera from Malawi (6).

## Discussion

To improve our understanding of naturally acquired immunity after gametocyte exposure, we assessed antibody responses to antigens in parasite and gametocyte extracts, selected recombinant *P. falciparum* proteins and a large panel of gametocyte-enriched proteins in volunteers from CHMI cohorts with different exposures to gametocytes. We showed that antibody responses to sexual stages are induced after a single exposure to relatively low gametocyte densities (peak densities up to ~1,600 gametocytes/mL). The antibody response to sexual stage-specific proteins was higher in participants exposed to higher gametocyte densities and was observed later than the response to well-characterised asexual antibody responses. Furthermore, we identified a list of known and new antigens that elicit antibodies that are associated with gametocyte exposure.

A handful of studies aimed to identify antibody responses to gametocyte-specific antigens in naturally exposed individuals. Several seroepidemiological studies, limited to the well-known gametocyte antigens Pfs48/45 and Pfs230 (44, 45), demonstrated rapid and short-lived gametocyte-specific antibody responses (46, 47), which do not necessarily increase with age as responses to some asexual antigens do (44, 48). Skinner et al. analysed antibody responses in the sera of Malian children to a large panel of putatively gametocyte-specific antigens based on the first published *P. falciparum* proteome. Comparing responses before and after the malaria season, they identified high seroprevalence of antibodies to the proteins Pfs16, PF3D7_1346400, PF3D7_1024800 and PfMDV1, indicating that these may be important biomarkers of gametocyte exposure. More recently, Muthui et al. set out to analyse naturally acquired antibody responses to seven antigens selected based on potential gametocyte surface expression, including Pfs230, PfMDV1 and five previously uncharacterized gametocyte antigens (49). They demonstrated antibody responses to all seven antigens, and suggested that PF3D7_0303900, PF3D7_1314500, and PF3D7_0208800 may have potential as markers of high gametocytaemia. Though undoubtably useful, these studies were not designed to assess the immune response to incident infection or accurately validate serological biomarkers as indicators of prior gametocyte exposure. In field settings, such assessments are challenging and require longitudinal observations before and after infection with sensitive quantification of parasite and gametocyte exposure. Complementary to the studies in natural gametocyte exposure populations, CHMI provides a unique opportunity in which the absence of prior exposure is guaranteed, and parasitaemia and gametocytaemia are monitored with high precision to provide metrics for cumulative exposure to different parasite life stages. In our current study, all CHMI cohorts showed an overall increase in antibody responses after infection to antigens in asexual as well as gametocyte extracts. CHMI control cohorts were exposed to asexual parasites but probably not to mature gametocytes: while gametocyte commitment may have occurred (50) and, potentially, early-stage gametocytes may have developed, early treatment of volunteers with a curative regimen of atovaquone/proguanil upon thick smear positivity makes it very unlikely that gametocytes completed maturation. Nevertheless, responsiveness in our gametocyte ELISA in CHMI control cohorts is not unexpected since the majority of antigens are shared between parasite stages (12), and responses to crude gametocyte extract were previously shown to be a poor predictor of gametocyte carriage (51).

Importantly, responses against sexual stage specific antigens were highest in our cohort that was exposed to the highest gametocyte burden, with antibodies to Pfs48/45 and Pfs230 showing a statistically significant increase during follow-up in the BS Gct group. In this cohort, anti-Pfs48/45 antibody responses were also strongly associated with the preceding gametocyte biomass; this response was observed only at day 51 after CHMI, indicating a slight lag from peak gametocytaemia (c. day 20) to its commensurate response (not observed at day 36) compared to the peak in parasite density (day 8) and the observation of a response (from day 21). Given the relative sizes of the asexual and gametocyte biomass and their anticipated antigenic insults, these findings are broadly in line with expectations. It is noteworthy though that despite the relative scarcity of gametocytes, antibody responses to some sexual stage antigens (Pfs48/45-10C) were ranked higher than the majority of asexual antigens, indicating that the native Pfs48/45 protein is highly immunogenic. One unexpected finding was the antibody response to CSP after infection with blood stage parasites; we hypothesize that the abundance of low complexity, highly immunogenic repeat regions shared by CSP and some blood stage antigens may have resulted in a degree of cross-reactivity (52). Unexpected blood stage antigen reactivity has been observed previously after RTS’S vaccination (53).

Pfs48/45 and Pfs230 represent two well-described gametocyte antigens, but as there are hundreds of proteins predicted to be enriched or specifically expressed in gametocytes (12, 54), these two represent a tiny fraction of antigens that are likely to induce antibodies during gametocyte exposure. The ability to detect gametocyte-specific antibodies in the BS Gct cohort encouraged us to try to identify other gametocyte antigens that induced specific responses. We thus compared antibody responses to a large panel of gametocyte-enriched proteins on a protein microarray between the SPZ Gct and BS Gct cohorts. A long list of gene products (n=64) was identified for further analysis if they: 1) Showed significant antibody response to infection in the high (BS Gct) but not in the low (SPZ Gct) gametocyte cohort; or 2) Showed antibody responses in either the low or high Gct cohorts while having a negligible response in control CHMI cohorts. Encouragingly, we identified multiple antigens that are known to be gametocyte-specific, including Pfs16 (55), Pfg27 (55), Pf11-1 (56) and Pfg377 (57). Furthermore, there was considerable overlap with gametocyte proteins on the surface of infected red blood cells identified by Dantzler et al. (6), and with antigens that were identified as being associated with functional transmission reducing activity (39). Given that antibody responses in our CHMI cohort participants were low, as compared to individuals with rare functional anti-gametocyte immunity (39, 58), and as expected, based on a single relatively low infection burden, we did not expect or assess the functionality of responses but focused on the kinetics of antibody acquisition and the potential utility of responses as biomarkers of gametocyte exposure.

Our findings are based on detailed assessments of parasite exposure and antibody responses following a first encounter with *Plasmodium* parasites in a small number of volunteers. A limitation of this study is the relatively low gametocyte exposure with median peak densities of 1,304 (IQR 308 – 1,607) gametocytes/mL combined with a relatively short duration of exposure. Whilst these densities are similar to that observed in many asymptomatic infections in endemic settings, much higher densities and, in particular, much longer exposure to gametocytes can be observed in naturally infected individuals (59). As such, it is conceivable that our analysis did not identify all markers of epidemiologically relevant gametocyte carriage. A second limitation, as described above, is that we did not formally demonstrate absence of gametocyte exposure in control cohorts. If gametocytes had developed in control subjects, our comparisons between cohorts may have resulted in a conservative interpretation of unique gametocyte responses. A further limitation is the limited number of timepoints for immunological assessments. Control cohorts did not have a late timepoint (C+51) of sampling and we were unable to examine antibody longevity beyond day 51. Assessing the duration of detectable antibody titers, isotype and avidity beyond this timeframe, and whether re-infection will boost and/or change these responses will be valuable in the characterization of antibodies as biomarkers of infectivity and could have implications for the development of transmission blocking vaccines (TBV). Natural boosting of antibody responses has been noted as an advantage for TBVs that target pre-fertilisation gametocyte antigens. We found that all BS Gct participants showed an increase in antibodies to TBV candidate antigens Pfs48/45 and Pfs230, including the Pfs48/45-6C and Pfs230CMB fragments that are similar to TBV targets currently in human trials (Ref (60). and clinicaltrials.gov: NCT04862416). Our findings suggest relevant natural boosting of Pfs48/45 and Pfs230 antibody responses; we further report on the first distinct analysis of responses against full length Pfs48/45, as well as three Pfs48/45 fragments: 10C, 10N and 6C.

In conclusion, we found increased humoral responses to *P. falciparum* sexual stages after exposure to a single CHMI, irrespective of gametocyte densities. The cohort with highest gametocyte exposure showed more sexual-stage specific responses compared to the cohort exposed to low gametocytes, while overall parasite responses were higher in SPZ Gct. Using a protein microarray we identified potential gametocyte specific antigens.

## Data availability statement

The original contributions presented in the study are included in the article/supplementary material. Further inquiries can be directed to the corresponding author.

## Ethics statement

The studies involving human participants were reviewed and approved by the central committee on research involving human subjects (CCMO). The patients/participants provided their written informed consent to participate in this study.

## Author contributions

RJ, MA, WS, and TB conceived and designed the study. RJ, MA, WS, TO, ED, KT performed experiments and data analysis. WS and TB designed the microarray, with input from MM and KD. KKAT and RJ designed proteins used in the bead-based antibody assays. RN and RR constructed the protein microarray under supervision of PF. RJ, MA, TO and WS performed the data analysis, with contributions from MM, PN, KC and CD. RJ, MA, TO, WS and TB wrote the manuscript with input from all authors. All authors approved the final manuscript.

## Funding

This work was supported by a Sir Henry Wellcome fellowship (number 218676/Z/19/Z) from the Wellcome Trust (UK) awarded to WS. TB, RMdJ and MA are supported by a fellowship from the European Research Council (ERC-CoG 864180; QUANTUM). CD and TO are supported by a project grant from the Bill and Melinda Gates foundation (INDIE OPP1173572), and KKAT is supported by the Intellectual Ventures Global Good Fund and a UKRI Connecting Capabilities fund/Bloomsbury SET (CCF-17-7779). MM and PN are supported by a Programme grant from the UKRI Medical Research Council (MR/T016272/1) and Wellcome Center award 104111.

## Conflict of interest

The authors declare that the research was conducted in the absence of any commercial or financial relationships that could be construed as a potential conflict of interest.

## Publisher’s note

All claims expressed in this article are solely those of the authors and do not necessarily represent those of their affiliated organizations, or those of the publisher, the editors and the reviewers. Any product that may be evaluated in this article, or claim that may be made by its manufacturer, is not guaranteed or endorsed by the publisher.
