# Supplementary material for: The acquisition of humoral immune responses targeting Plasmodium falciparum sexual stages in controlled human malaria infections
Source: Front Immunol. 2022 Jul 18;13:930956. doi: 10.3389/fimmu.2022.930956 (PMC9339717; doi:10.3389/fimmu.2022.930956)
Supplement: Supplementary file 1 [file DataSheet_1.docx]

**Supplemental table 1. Recombinant antigens used in the luminex bead-based antibody quantification assay**

| **Gene ID** | **Acronym** | **Description** | **Location** | **Expression tag** | **Strain** | **Reference** |
| --- | --- | --- | --- | --- | --- | --- |
| PF3D7_0304600 | CSP | Sporozoite surface. Component of RTS,S vaccine | Sporozoite | n/a | 3D7 | ^1^ |
| PF3D7_1301600 | EBA140 RIII-V | Erythrocyte binding antigen 140; erythrocyte invasion | Apical organelles, micronemes | GST | 3D7 | ^2^ |
| PF3D7_0731500 | EBA175RII_F2 | Erythrocyte binding antigen 175; RBC binding region via glycophorin A | Apical tip | GST | 3D7 | ^2^ |
| PF3D7_0102500 | EBA181 RIII-V | Erythrocyte binding antigen 181; involved in erythrocyte invasion | Apical tip | GST | 3D7 | ^2^ |
| PF3D7_0532100 | Etramp 5 Ag 1 | Early transcribed membrane protein. Integral PVM protein | iRBC, PVM | GST | 3D7 | LSHTM |
| PF3D7_1035300 | GLURP R2 | Glutamate rich protein R2 | Merozoite Surface | n/a | F32 | ^3^ |
| PF3D7_0501100.1 | HSP40 Ag 1 | Heat shock protein 40 | iRBC | GST | 3D7 | LSHTM |
| PF3D7_0206800 | MSP2 CH150/9 | CH150/9 allele of MSP2. Full-length. | Merozoite surface | GST | CH150/9 | ^4^ |
| PF3D7_0930300 | MSP1-19 | 19kDa fragment of MSP1 molecule. | Merozoite surface | GST | Wellcome | ^5^ |
| PF3D7_0206800 | MSP2 Dd2 | Dd2 allele of MSP2. Full-length. | Merozoite surface | GST | DD2 | ^6^ |
| PF3D7_1021800 | PfSEA-1 | Schizont egress antigen | iRBC | GST | 3D7 | LSHTM |
| PF3D7_0501300 | SBP1 | Skeleton-binding protein; translocation of PfEMP1 to RBC surface via Maurer's cleft | iRBC | GST | 3D7 | LSHTM |
| PF3D7_1133400 | AMA1 | Apical membrane antigen 1 | Micronemes | His | FVO | ^7^ |
| PF3D7_1335400 | Rh2_2030 | Reticulocyte-binding protein homolog 2; involved in erythrocyte invasion | Merozoites; Rhoptries | GST | 3D7 | ^8^ |
| PF3D7_0424200 | Rh4.2 | Reticulocyte-binding protein homolog 4; involved in erythrocyte invasion | Merozoites; Rhoptries | GST | 3D7 | ^9^ |
| PF3D7_0424100 | Rh5.1 | Reticulocyte-binding protein homolog 5; involved in erythrocyte invasion | Merozoites; Rhoptries | His | 3D7 | ^10^ |
| PF3D7_1346700 | Pfs48/45-10C | Gametocyte/gamete surface protein, 10C fragment | Mature gametocytes/gametes | His | 3D7 | Radboud |
| PF3D7_1346700 | Pfs48/45-10N | Gametocyte/gamete surface protein, 10N fragment | Mature gametocytes/gametes | His | 3D7 | Radboud |
| PF3D7_1346700 | Pfs48/45-6C | Gametocyte/gamete surface protein, 6C fragment | Mature gametocytes/gametes | His | 3D7 | Radboud |
| PF3D7_1346700 | Pfs48/45-Full length | Gametocyte/gamete surface protein, Full Length | Mature gametocytes/gametes | His | 3D7 | Radboud |
| PF3D7_0209000 | Pfs230-CMB | Gametocyte/gamete surface protein, CMB fragment | Mature gametocytes/gametes | His | 3D7 | ^11^ |

Radboud: Produced in house at Radboudumc; LSHTM: Produced in house at LSHTM

**Supplemental table 2. Selection criteria for genes expressed as proteins on the gametocyte protein microarray**

| **Criteria ID** | **Genes matching criteria** | ***Genes added*** | **Gene cumulative total** | **Criteria for inclusion** | **Simplified criteria** |
| --- | --- | --- | --- | --- | --- |
| **1** | 372 | *372* | 372 | Gametocyte protein score >0 PLUS (presence of TM/SP/GO term indicating membrane OR surface expression (excluding mitochondrial proteins)) | Protein moderately upregulated in gametocytes, and indication of membrane expression (excluding mitochondrial) |
| **2** | 36 | *35* | 407 | Zero protein evidence in any proteomic analysis, or evidence of translational repression in literature PLUS high gametocyte transcript score (>9.63) PLUS (presence of tm/sp/go term indicating membrane OR surface expression (excluding mitochondrial proteins)) | Zero protein scores but gametocyte specific transcript (evidence of failure to detect protein, and/or mRNA storage/translational repression) |
| **3** | 60 | *29* | 436 | Gametocyte protein score >-10 (some evidence) PLUS (presence of GPI anchor ^12^ OR predicated export protein (Public domain). | Presence of protein in gametocytes, plus GPI anchor, or export association |
| **4** | 30 | *7* | 443 | Putatively exported by and specific to early or late gametocytes ^13^ | Additional exported proteins specific to early gametocytogenesis |
| **5** | 107 | *75* | 518 | Gametocyte protein score of >9.69, regardless of protein structure/function, and an average peptide score of 9 in each gametocyte proteome database | Highly upregulated in gametocytes regardless of structure/function |
| **6** | 43 | *13* | 531 | Gametocyte specific proteins ^14^ seropositive in field sera in >50% of samples after transmission season OR with >20% seroprevalence increase after transmission season ^15^ | Gametocyte specific in the first gametocyte proteomic analysis ^14^ with evidence of immune recognition in Skinner et al ^15^ (>50% samples after transmission season or >20% increase) |
| **7** | 41 | *7* | 538 | Gold standard gametocyte protein in list used to generate transcript and protein score | Gold standard gametocyte proteins |
| **8** | 37 | *23* | 561 | *A prioiri* selection. Markers of sexual stage exposure, TBV candidates, 6-cys proteins, proteins implicated in gamete fertility, and markers of asexual exposure | *A priori* selection: markers of asexual and sexual stage, vaccine candidates |
| **9** | 45 | *19* | 580 | Inclusion based on correlation with transmission blocking immunity ^16^ | Inclusion based on association with transmission blocking immunity in our earlier work ^16^ |
| **10** | 47 | *20* | **600** | Inclusion based on recognition by mice/immune sera and presence on giRBC surface ^17^ | Inclusion based on presence and recognition on giRBC surface (early gametocytes) ^17^. |

Selection was made as follows, initially prioritizing proteins with characteristics necessary for surface level expression and less conservative gametocyte specificity (selection for involvement in TRI), before including proteins with greater gametocyte specificity regardless of their function (selection of markers of exposure): **1.** Proteins with evidence for presence or enrichment in gametocytes (score >0) PLUS presence of transmembrane domains (tm), signal peptides (sp), or GO term indicating membrane or surface expression (excluding mitochondrial proteins) [n=372] **2.** Proteins without unique peptides in any proteomic analysis, or evidence of translational repression in literature **^18^** PLUS high gametocyte transcript score (>9.63), PLUS presence of TM/SP/GO term indicating membrane or surface expression (excluding mitochondrial proteins) [n=36, cumulative total = 407] **3.** Gametocyte protein score >-10 (some evidence of gametocyte expression) PLUS presence of GPI anchor ^12^ or predicted export protein (PlasmoDB) [n=60, cum. total = 436] Proteins putatively exported by and specific to early or late gametocytes ^13^ n=30, cum. total = 443] **5.** Gametocyte protein score of >9.69 (Gametocyte enriched), regardless of protein structure/function, and an average peptide score of 9 in each of the gametocyte databases [n=107, cum. total = 518) 6. Gametocyte specific proteins from early literature ^14,19^. PLUS seropositive in field sera in >50% of samples after transmission season or with >20% sero-prevalence increase after transmission season ^15^ (n=43, cum. total 451) **7.** Gold standard gametocyte proteins in list used to generate transcript and protein score [n=41, cum. total = 538) **8.** *A priori* protein selection. Markers of sexual stage exposure, transmission blocking vaccine candidates, 6-cys proteins, proteins implicated in gamete fertility, asexual markers etc. (n=37, cum. total = 561) **9.** Inclusion based on previous gametocyte array analysis ^16^ of differently reactive proteins in naturally occurring transmission blockers [n=45, cum. total = 580), 10. Inclusion based on association with the giRBC surface of immature gametocytes ^17^ [n=47, cumulative total and total number of IVTT targets on array **= 600**]. Of the 600 proteins selected for inclusion, 568 were successfully expressed and printed on the array (943 IVTT protein targets).

**Supplemental table 3 (in Supplemental xlsx file). Differences in net MFI for antibody responses assessed in bead based assay between baseline (C-1) and subsequent timepoints.** Differences were tested with paired t-tests to account for repeated measures and adjust for variable baseline antibody reactivities. Values are p-values unadjusted for multiple comparisons (<0.05=red). Values below the Bonferroni adjusted threshold for significance are highlighted in green. All differences are positive (higher response after baseline). Responses at day C+7/9 were not significantly increased over baseline for any antigen.

**Supplementary Table 4. Position of sexual stage antigens in ranked median magnitude of response amongst all antigens.** Colour scale indicates the number of positions moved up (green), down (red), or if there is no change (white), between C+36 and C+51 for the CHMI cohorts.

|  | **SPZ Gct** | | **BS Gct** | | **SPZ Control** | **BS Control** |
| --- | --- | --- | --- | --- | --- | --- |
|  | **C+36** | **C+51** | **C+36** | **C+51** | **C+35** | **C+35** |
| **Pfs48/45-10C** | 6 | 5 | 8 | 3 | 7 | 4 |
| **Pfs48/45-10N** | 11 | 9 | 10 | 6 | 12 | 10 |
| **Pfs48/45-6C** | 7 | 7 | 9 | 9 | 6 | 7 |
| **Pfs48/45 full length** | 9 | 10 | 13 | 8 | 9 | 5 |
| **Pfs230-CMB** | 13 | 14 | 14 | 14 | 10 | 8 |

**Supplemental table 5. Correlation between total parasite and gametocyte AUC and antibody response to all recombinant antigens.** R^2^ and p-value are from Spearman’s rank order correlation. Antibody assay data used were log2 MFI ratios of response over each individual’s baseline. Parasite AUC was log transformed for analysis. Values are p-values unadjusted for multiple comparisons (<0.05=red). Values below the Bonferroni adjusted threshold for significance are highlighted in green. Serology data are all from the latest timepoint observed in the trials (C+51). AUC: Area under the curve.

|  | **Total parasite AUC** | | | | **Gametocyte AUC** | | | |
| --- | --- | --- | --- | --- | --- | --- | --- | --- |
|  | **SPZ Gct** | | **BS Gct** | | **SPZ Gct** | | **BS Gct** | |
|  | ***R^2^*** | ***p*** | ***R^2^*** | ***p*** | ***R^2^*** | ***p*** | ***R^2^*** | ***p*** |
| **CSP** | 0.00016 | 0.97 | 0.11 | 0.3 | 0.057 | 0.45 | 0.037 | 0.55 |
| **EBA140.RIII.V** | 0.0084 | 0.78 | 0.077 | 0.38 | 0.014 | 0.71 | 0.15 | 0.22 |
| **EBA175.RIII.V** | 0.06 | 0.44 | 0.099 | 0.32 | 0.014 | 0.71 | 0.2 | 0.14 |
| **EBA181.RIII.V** | 0.16 | 0.2 | 0.0041 | 0.84 | 0.073 | 0.4 | 0.0034 | 0.86 |
| **Etramp5.Ag1** | 0.0015 | 0.9 | 0.052 | 0.48 | 0.0061 | 0.81 | 0.068 | 0.41 |
| **GLURP.R2** | 0.057 | 0.46 | 0.0066 | 0.8 | 0.0071 | 0.79 | 0.045 | 0.51 |
| **HSP40.Ag1** | 0.18 | 0.17 | 0.003 | 0.87 | 0.26 | 0.088 | 0.01 | 0.76 |
| **MSP2.CH150** | 0.09 | 0.34 | 0.0013 | 0.91 | 0.22 | 0.12 | 5.20E-06 | 0.99 |
| **MSP2.Dd2** | 0.27 | 0.081 | 0.062 | 0.44 | 0.15 | 0.21 | 0.11 | 0.29 |
| **PfAMA1** | 0.081 | 0.37 | 0.17 | 0.19 | 4.90E-05 | 0.98 | 0.34 | 0.048 |
| **PfMSP119** | 0.039 | 0.54 | 0.64 | 0.0017 | 0.018 | 0.68 | 0.56 | 0.005 |
| **Pfs230.CMB** | 0.37 | 0.035 | 0.019 | 0.67 | 0.41 | 0.025 | 0.0045 | 0.84 |
| **Pfs.48.45.10C** | 0.015 | 0.7 | 0.6 | 0.003 | 0.24 | 0.11 | 0.64 | 0.0019 |
| **Pfs.48.45.10N** | 0.018 | 0.68 | 0.33 | 0.053 | 0.41 | 0.024 | 0.5 | 0.011 |
| **Pfs.48.45.6C** | 0.11 | 0.29 | 0.27 | 0.083 | 0.18 | 0.17 | 0.21 | 0.13 |
| **Pfs.48.45.full.length** | 0.0046 | 0.83 | 0.12 | 0.26 | 0.17 | 0.19 | 0.18 | 0.17 |
| **PfSEA** | 0.16 | 0.2 | 0.082 | 0.37 | 0.093 | 0.34 | 0.049 | 0.49 |
| **Rh2.2030** | 0.29 | 0.073 | 0.02 | 0.66 | 0.14 | 0.23 | 0.063 | 0.43 |
| **Rh4.2** | 0.0047 | 0.83 | 0.053 | 0.47 | 0.027 | 0.61 | 0.027 | 0.61 |
| **Rh5.1** | 0.23 | 0.12 | 0.0021 | 0.89 | 0.18 | 0.17 | 0.0022 | 0.88 |
| **SBP1** | 0.11 | 0.29 | 0.21 | 0.13 | 0.15 | 0.21 | 0.26 | 0.092 |

**Supplemental table 6.** **Antibody targets with differential response between cohorts or over the time course of infection (in Supplemental xlsx file).** Key in tab 2. Proteins were classified based on protein expression profiles, using raw peptide counts obtained from proteomics data annotations on PlasmoDB (plasmodb.org version 56, 15 Feb 2022). Data originally published in Silvestrini, F., et al., Mol Cell Proteomics, 2010; Florens, L., et al., Mol Biochem Parasitol, 2004; Lasonder, E., et al., Nucleic Acids Res, 2016; Oehring, S.C., et al., Genome Biol, 2012.

**
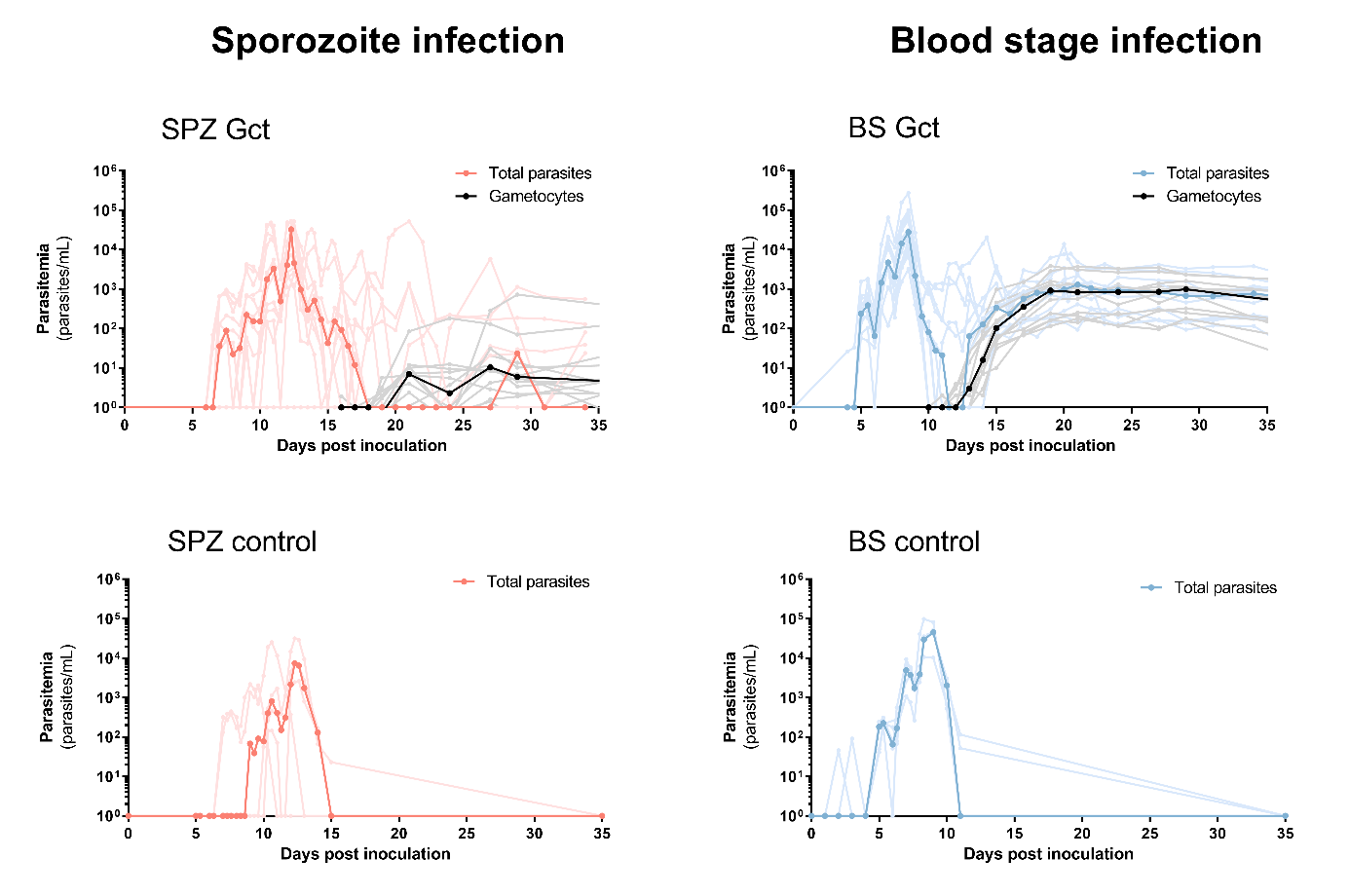
**

**Supplemental figure 1. Parasite densities over time in the CHMI cohorts.** Total parasite densities were determined by 18S quantitative polymerase chain reaction. Shaded lines represent data from individual participants, bold lines indicate the median values. Gametocyte densities are the sum of ccp4 and pfmget quantitative reverse-transcription polymerase chain reaction (qRT-PCR) data. Gray lines represent data from individual participants and the black line the median gametocyte density.

***
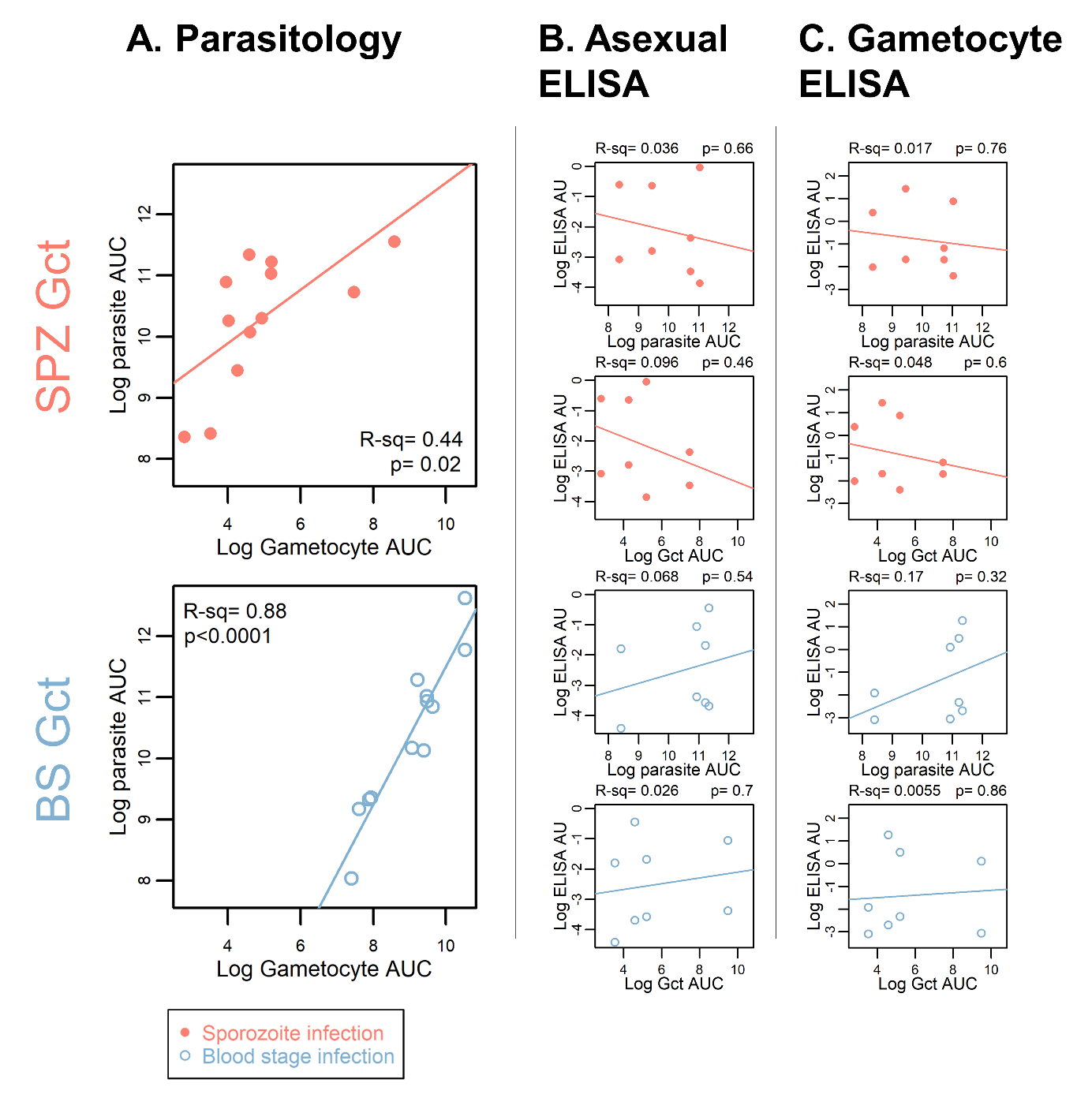
***

**Supplemental figure 2. Participant parasite exposure and antibody response to crude parasite extracts.** In all plots, red (top) denote Gct A (sporozoite infection) and blue (bottom) denotes Gct B (Asexual infection). **A.** The association of total parasite and gametocyte density areas under the curve (AUC) **B.** The association of total parasite AUC and gametocyte AUC with antibody response to asexual parasite extract. **C.** The association of total parasite AUC and gametocyte AUC with antibody response to gametocyte extract. r = Spearman’s Rho, p = p-value from Spearman’s rank test.

***
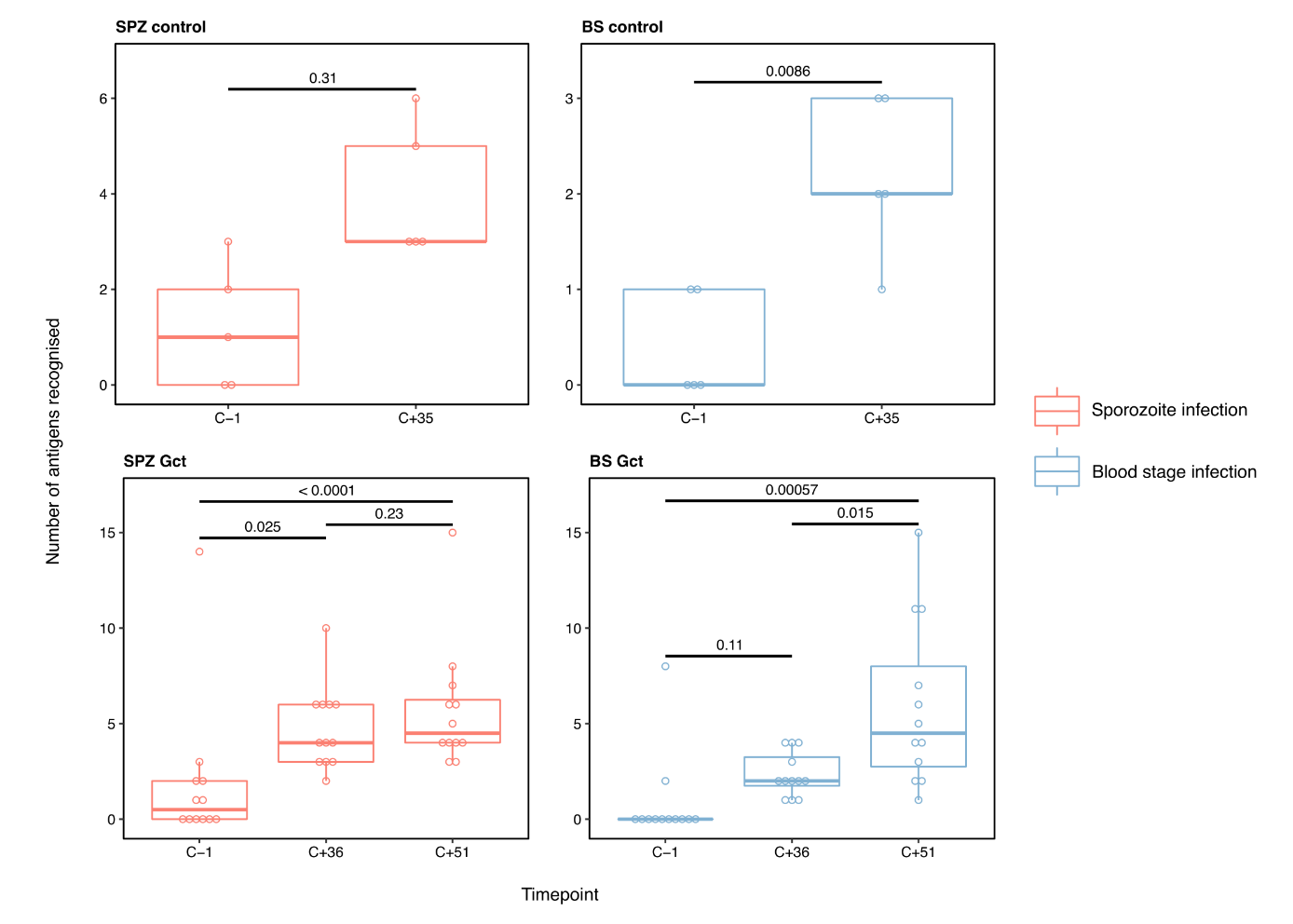
***

**Supplemental figure 3. Cumulative breadth of positive antibody responses to recombinant antigens.** Participants were considered to recognise an antigen (i.e. be seropositive) if background corrected MFI values were greater than the mean background corrected MFI plus 2 standard deviations of all individuals at baseline. In all plots red boxes indicate mosquito bite (sporozoite) infection cohorts (SPZ Gct, SPZ control) and blue boxes denote asexual parasite infection (BS Gct, BS control).


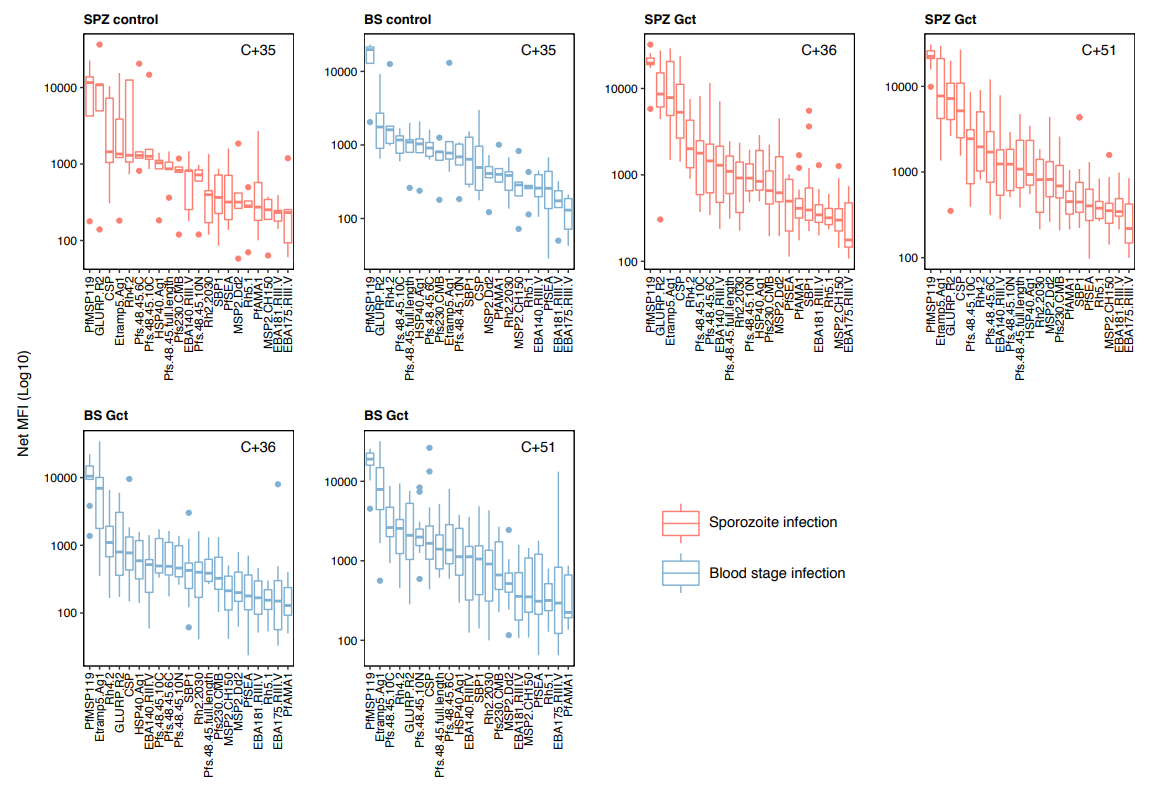


**Supplemental figure 4. Magnitude of antibody response to all recombinant antigens.** Background corrected MFI values for all antigens, presented on a log10 scale on the y-axis, ordered by median response (strong bars) on the x-axis. In all plots red boxes indicate mosquito bite (sporozoite) infection cohorts (SPZ Gct, SPZ control) and blue boxes denote asexual parasite infection (BS Gct, BS control).


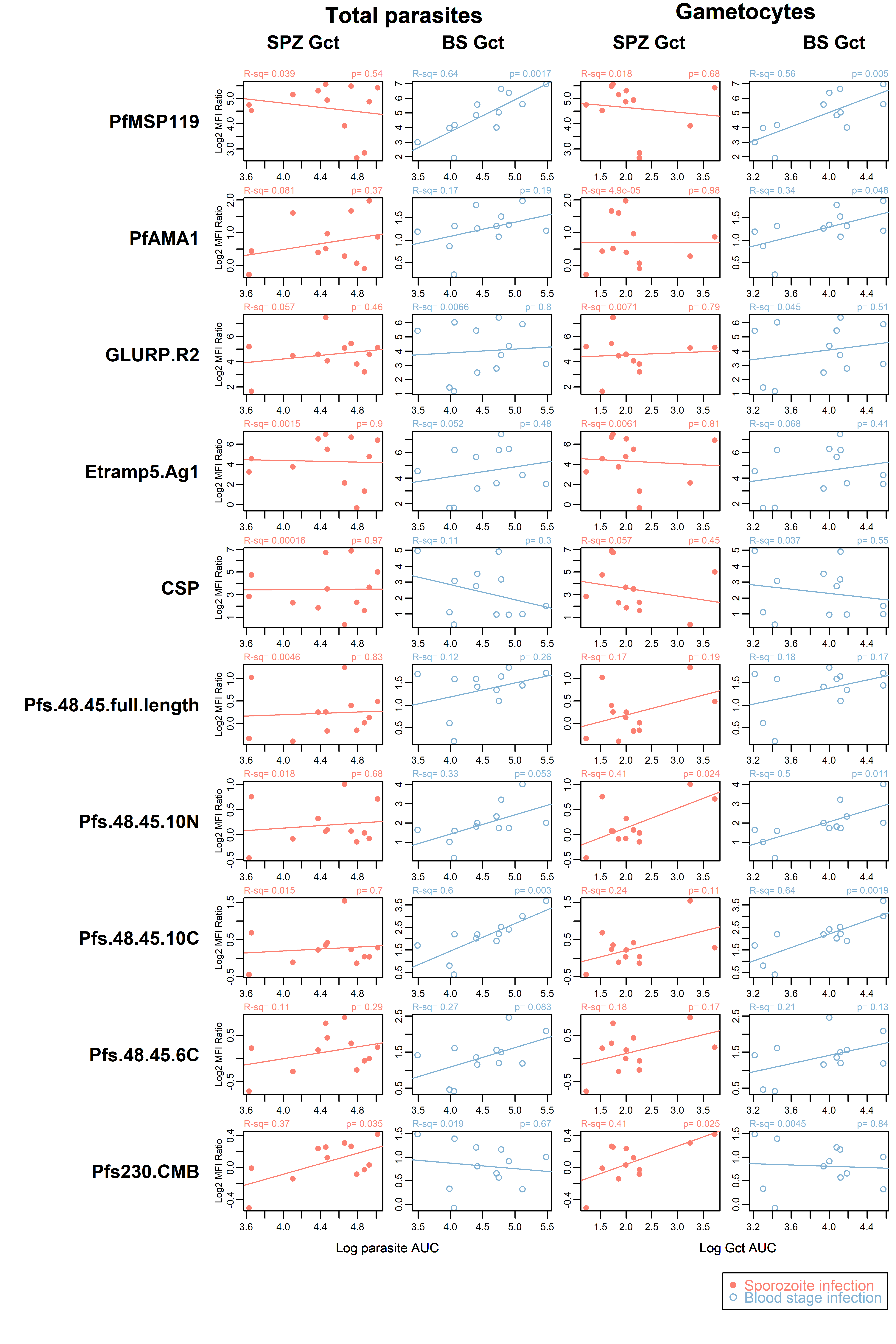


**Supplemental figure 5. Correlation between total parasite and gametocyte AUC and antibody response to selected antigens.** R^2^ and p-value are from Spearman’s rank order correlation, with trend line from simple linear regression. In all plots, red solid circles denote mosquito bite (sporozoite) infection cohorts (SPZ Gct, SPZ control) and blue hollow circles denote asexual parasite infection (BS Gct, BS control). Antibody assay data are presented as log2 MFI ratios of response over each individual’s baseline.


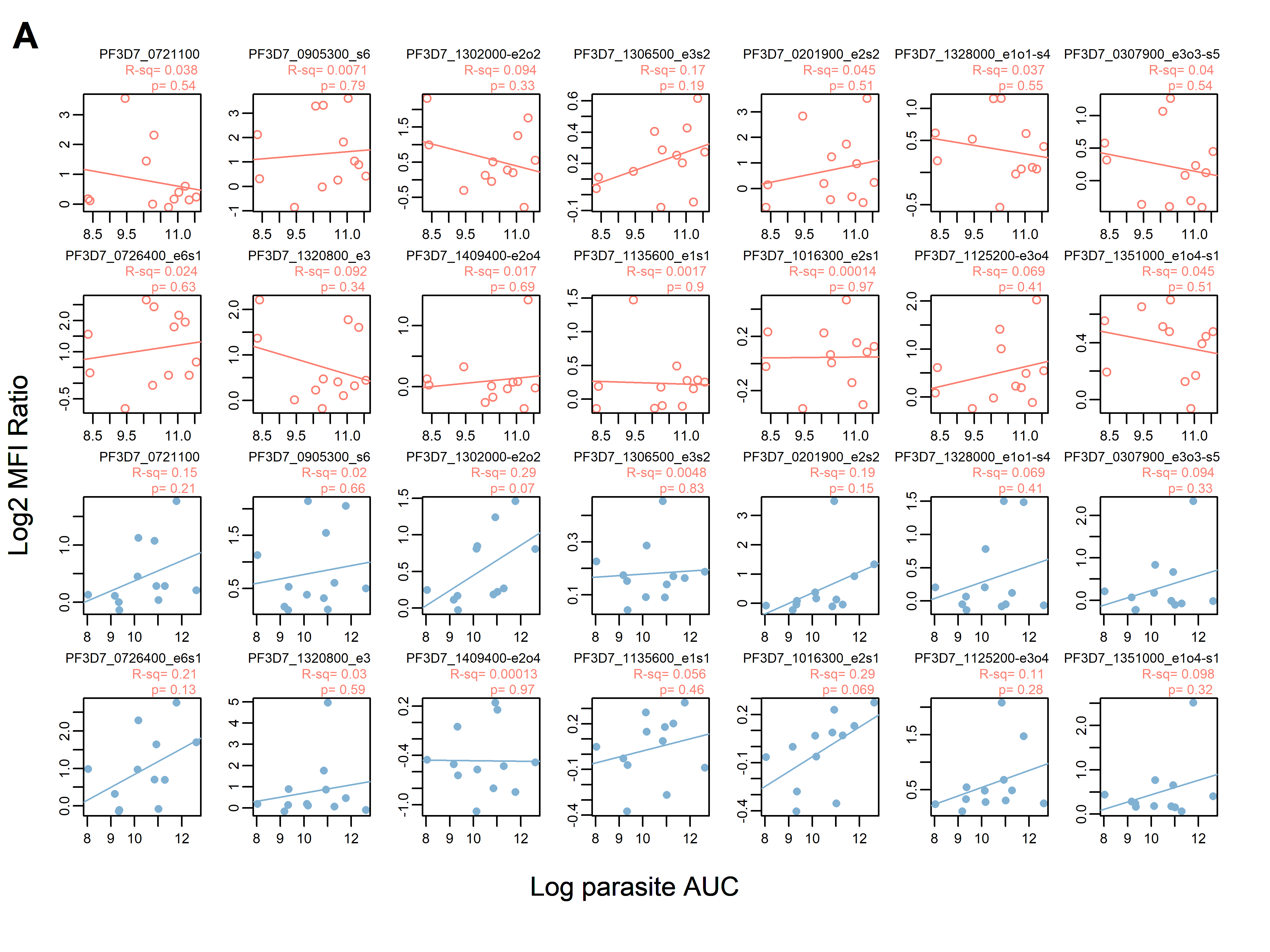


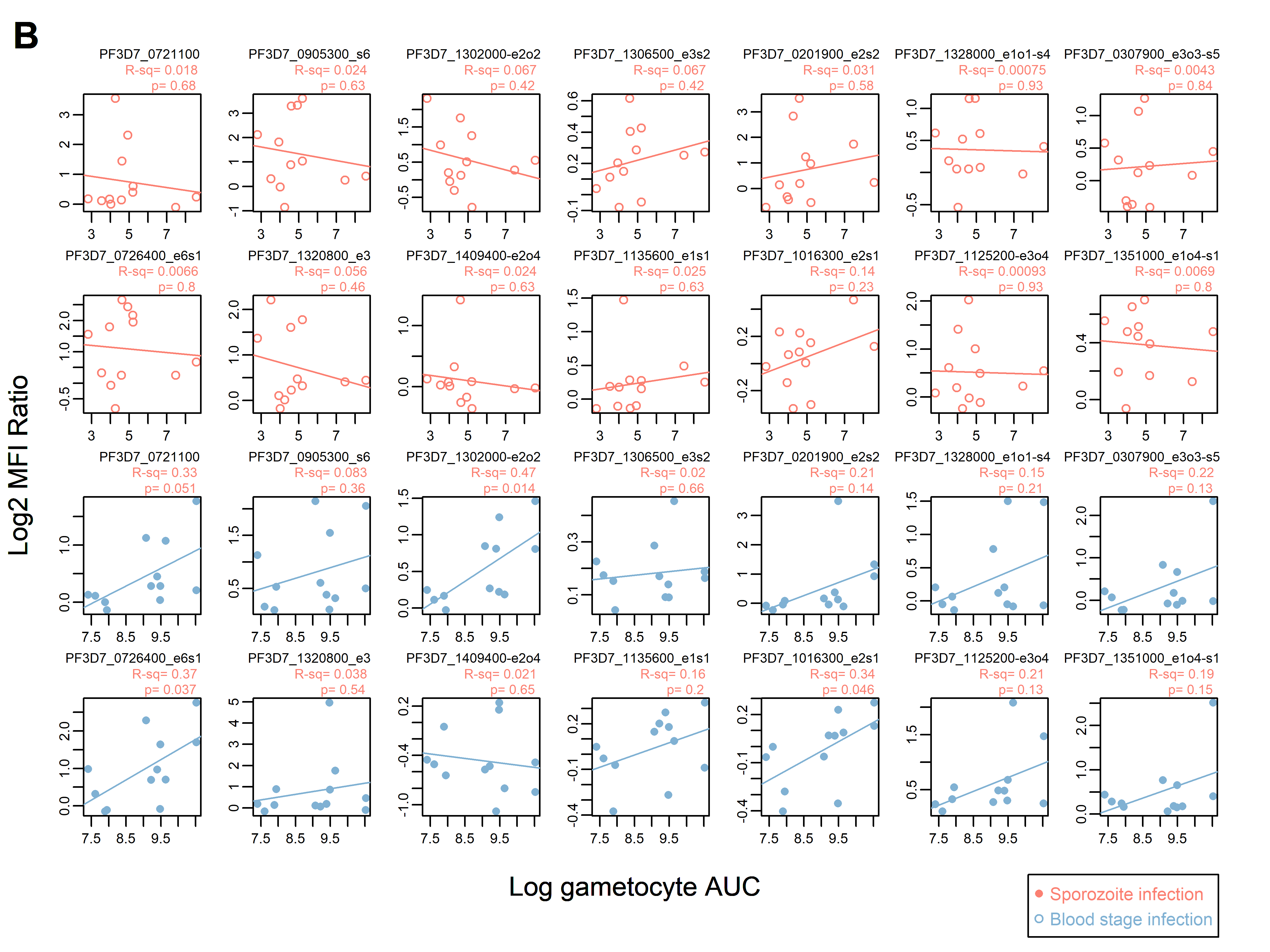


**Supplemental figure 6. Correlation between asexual and gametocyte AUC and antibody responses to a selection of potential antibody biomarkers of gametocyte exposure. A.** Correlation of antibody response to curated array targets and total parasite AUC. **B.** Correlation of antibody response to curated array targets and gametocyte AUC. R^2^ and p-value are from Spearman’s rank order correlation, with trend line from simple linear regression. In all plots, red solid circles denote SPZ Gct and blue hollow circles denote BS Gct. Array data are presented as log2 MFI ratios as described in the methods.

1 Kastenmuller, K. *et al.* Full-length Plasmodium falciparum circumsporozoite protein administered with long-chain poly(I.C) or the Toll-like receptor 4 agonist glucopyranosyl lipid adjuvant-stable emulsion elicits potent antibody and CD4+ T cell immunity and protection in mice. *Infect Immun* **81**, 789-800, doi:10.1128/IAI.01108-12 (2013).

2 Richards, J. S. *et al.* Association between naturally acquired antibodies to erythrocyte-binding antigens of Plasmodium falciparum and protection from malaria and high-density parasitemia. *Clin Infect Dis* **51**, e50-60, doi:10.1086/656413 (2010).

3 Theisen, M. *et al.* Antigenicity and immunogenicity of recombinant glutamate-rich protein of Plasmodium falciparum expressed in Escherichia coli. *Clin Diagn Lab Immunol* **2**, 30-34, doi:10.1128/cdli.2.1.30-34.1995 (1995).

4 Polley, S. D. *et al.* High levels of serum antibodies to merozoite surface protein 2 of Plasmodium falciparum are associated with reduced risk of clinical malaria in coastal Kenya. *Vaccine* **24**, 4233-4246, doi:10.1016/j.vaccine.2005.06.030 (2006).

5 Burghaus, P. A. & Holder, A. A. Expression of the 19-kilodalton carboxy-terminal fragment of the Plasmodium falciparum merozoite surface protein-1 in Escherichia coli as a correctly folded protein. *Mol Biochem Parasitol* **64**, 165-169, doi:10.1016/0166-6851(94)90144-9 (1994).

6 Taylor, R. R. *et al.* Human antibody response to Plasmodium falciparum merozoite surface protein 2 is serogroup specific and predominantly of the immunoglobulin G3 subclass. *Infect Immun* **63**, 4382-4388, doi:10.1128/iai.63.11.4382-4388.1995 (1995).

7 Collins, C. R. *et al.* Fine mapping of an epitope recognized by an invasion-inhibitory monoclonal antibody on the malaria vaccine candidate apical membrane antigen 1. *J Biol Chem* **282**, 7431-7441, doi:10.1074/jbc.M610562200 (2007).

8 Triglia, T. *et al.* Reticulocyte-binding protein homologue 1 is required for sialic acid-dependent invasion into human erythrocytes by Plasmodium falciparum. *Mol Microbiol* **55**, 162-174, doi:10.1111/j.1365-2958.2004.04388.x (2005).

9 Reiling, L. *et al.* The Plasmodium falciparum erythrocyte invasion ligand Pfrh4 as a target of functional and protective human antibodies against malaria. *PLoS One* **7**, e45253, doi:10.1371/journal.pone.0045253 (2012).

10 Hjerrild, K. A. *et al.* Production of full-length soluble Plasmodium falciparum RH5 protein vaccine using a Drosophila melanogaster Schneider 2 stable cell line system. *Sci Rep* **6**, 30357, doi:10.1038/srep30357 (2016).

11 Farrance, C. E. *et al.* A plant-produced Pfs230 vaccine candidate blocks transmission of Plasmodium falciparum. *Clin Vaccine Immunol* **18**, 1351-1357, doi:10.1128/CVI.05105-11 (2011).

12 Gilson, P. R. *et al.* Identification and stoichiometry of glycosylphosphatidylinositol-anchored membrane proteins of the human malaria parasite Plasmodium falciparum. *Mol Cell Proteomics* **5**, 1286-1299, doi:10.1074/mcp.M600035-MCP200 (2006).

13 Silvestrini, F. *et al.* Protein export marks the early phase of gametocytogenesis of the human malaria parasite Plasmodium falciparum. *Mol Cell Proteomics* **9**, 1437-1448, doi:10.1074/mcp.M900479-MCP200 (2010).

14 Florens, L. *et al.* A proteomic view of the Plasmodium falciparum life cycle. *Nature* **419**, 520-526, doi:10.1038/nature01107 (2002).

15 Skinner, J. *et al.* Plasmodium falciparum Gametocyte-Specific Antibody Profiling Reveals Boosting through Natural Infection and Identifies Potential Markers of Gametocyte Exposure. *Infect Immun* **83**, 4229-4236, doi:10.1128/IAI.00644-15 (2015).

16 Stone, W. J. R. *et al.* Unravelling the immune signature of Plasmodium falciparum transmission-reducing immunity. *Nat Commun* **9**, 558, doi:10.1038/s41467-017-02646-2 (2018).

17 Dantzler, K. W. *et al.* Naturally acquired immunity against immature Plasmodium falciparum gametocytes. *Sci Transl Med* **11**, doi:10.1126/scitranslmed.aav3963 (2019).

18 Lasonder, E. *et al.* Integrated transcriptomic and proteomic analyses of P. falciparum gametocytes: molecular insight into sex-specific processes and translational repression. *Nucleic Acids Res* **44**, 6087-6101, doi:10.1093/nar/gkw536 (2016).

19 Hall, N. *et al.* A comprehensive survey of the Plasmodium life cycle by genomic, transcriptomic, and proteomic analyses. *Science* **307**, 82-86, doi:10.1126/science.1103717 (2005).

**Supplementary references**
